# Immersive Virtual Reality for Patient-Specific Preoperative Planning: A Systematic Review

**DOI:** 10.1177/15533506221143235

**Published:** 2022-11-30

**Authors:** Lucy Lan, Randi Q. Mao, Reva Y. Qiu, Jeffrey Kay, Darren de Sa

**Affiliations:** 1 Michael G. DeGroote School of Medicine, 62703 McMaster University, Hamilton, ON, Canada; 2 Division of Orthopaedic Surgery, Department of Surgery, 62703 McMaster University, Hamilton, ON, Canada

**Keywords:** surgical education, simulation, radiologist, image guided surgery, ergonomics and/or human factors study

## Abstract

*Background.* Immersive virtual reality (iVR) facilitates surgical decision-making by enabling surgeons to interact with complex anatomic structures in realistic 3-dimensional environments. With emerging interest in its applications, its effects on patients and providers should be clarified. This systematic review examines the current literature on iVR for patient-specific preoperative planning. *Materials and Methods.* A literature search was performed on five databases for publications from January 1, 2000 through March 21, 2021. Primary studies on the use of iVR simulators by surgeons at any level of training for patient-specific preoperative planning were eligible. Two reviewers independently screened titles, abstracts, and full texts, extracted data, and assessed quality using the Quality Assessment Tool for Studies with Diverse Designs (QATSDD). Results were qualitatively synthesized, and descriptive statistics were calculated. *Results.* The systematic search yielded 2,555 studies in total, with 24 full-texts subsequently included for qualitative synthesis, representing 264 medical personnel and 460 patients. Neurosurgery was the most frequently represented discipline (10/24; 42%). Preoperative iVR did not significantly improve patient-specific outcomes of operative time, blood loss, complications, and length of stay, but may decrease fluoroscopy time. In contrast, iVR improved surgeon-specific outcomes of surgical strategy, anatomy visualization, and confidence. Validity, reliability, and feasibility of patient-specific iVR models were assessed**.** The mean QATSDD score of included studies was 32.9%. *Conclusions.* Immersive VR improves surgeon experiences of preoperative planning, with minimal evidence for impact on short-term patient outcomes. Future work should focus on high-quality studies investigating long-term patient outcomes, and utility of preoperative iVR for trainees.

## Introduction

As operative procedures become increasingly complex, so too does surgical decision-making. Patient-specific preoperative planning enables surgeons to optimize approaches and anticipate difficulties, allowing for improved patient safety and decreased operative duration.^1^ Cross-sectional medical imaging modalities such as computed tomography (CT) and magnetic resonance imaging (MRI) were first introduced in the 1970s, providing surgeons with the capacity to better diagnose and evaluate anatomic structures with 2-dimensional (2D) pictures.^2,3^ However, these imaging techniques were unable to recreate complex 3-dimensional (3D) visualizations of anatomy as would be viewed in the operating room. Consequently, surgeons had to spend cognitive resources to translate these segmented 2D views into 3D mental models.^4,5^

3D reconstructions of cross-sectional imaging have been made widely available in radiologic suites to alleviate the cognitive load of image interpretation.^6^ However, surgeons are unable to manipulate components within these visualizations, limiting its applications in preoperative planning and training. Over recent years, patient-specific 3D-printed models have been adopted for greater user interactivity and rehearsal prior to surgery. These models can help surgeons better visualize the surgical anatomy, demonstrating improvements in outcomes such as operative time, intraoperative blood loss, and fluoroscopy usage.^3,7^ Although 3D printing is an exciting technology for preoperative planning, it is limited by high costs, unique personnel requirements, long production times, and restrictions in recreating soft tissue structures with high anatomical detail.^3^ A suitable alternative lies within virtual reality (VR) technology.

Virtual reality has the power to render 2D images into a 3D stereoscopic computer-generated environment.^8^ Immersive VR (iVR) expands upon conventional VR (where anatomical details are displayed on computer screens) by projecting the 3D environment onto a head-mounted display (HMD), allowing for 360° of visual immersion and real-time manipulation of virtual items. Immersive VR offers high fidelity visualizations and operates on portable, low cost, commercially-available hardware.^9^ Recently, iVR has been applied in many surgical education contexts, including anatomy instruction, intraoperative communication, surgical skills training, and the topic of this paper, preoperative planning.^10–12^ Along with its ability to potentially improve patient-important intraoperative and postoperative outcomes, the visuospatial skills gained from iVR may be translatable to the OR environment and contribute to surgeon-important outcomes, such as satisfaction and anatomy comprehension.^8,13^

This systematic review aims to summarize the use of iVR for patient-specific preoperative planning and characterize its impacts on both quantitative and qualitative patient- and surgeon-specific outcomes. We also attempt to identify the strengths, shortcomings, and future directions for this emerging technology.

## Methods

This Study Adhered to the Preferred Reporting Items for Systematic Reviews and Meta-Analyses Statement (PRISMA).^14,15^

### Search Strategy

A systematic literature search for relevant English language articles was conducted using MEDLINE, EMBASE, CENTRAL, Web of Science, and Scopus. The results were limited to publications from January 1, 2000 through March 21, 2021. We used the following keywords: (Virtual Reality OR VR OR iVR OR “Head-mounted” OR “Head mounted” OR “Face-mounted” OR “Face mounted”) AND (Surgical Procedures, Operative OR Surg*) AND (Preop* OR Pre-op* OR “Pre Op*” OR Pre-surgical OR Pre-surgery OR Presurgical OR Presurgery OR “Pre Surg*” OR Patient-specific OR “Patient specific) AND (Plan* OR Train* OR Practi* OR Warm-up* OR “Warm up*”). Supplementary Table S1 lists the full search strategy for each database. We performed a hand search of related articles on Google Scholar and references of included studies for additional eligible studies. We did not specifically search grey literature or conference proceedings.

#### Eligibility Criteria

Eligible studies 1) included medical personnel at all levels (including undergraduate, postgraduate and staff physician levels) for 2) patient-specific preoperative planning using data acquired from any diagnostic imaging modality displayed through 3) an iVR simulator. We defined surgery as a therapeutic or diagnostic procedure involving incision of tissue (e.g., skin, fat, bone) in an operating room setting.^16^ We excluded studies that involved 1) non-medical personnel (e.g., dentists, nurses), 2) non-surgical procedures (e.g., endoscopy, interventional radiology), 3) simulators that used generic anatomic data (i.e., not patient-specific imaging) and/or 4) applications other than preoperative planning (e.g., anatomy education, non-patient-specific surgical skills training, intraoperative anatomy visualization). We also excluded reviews, editorials, opinion-based articles, and abstracts without full-texts available.

#### Screening and Study Selection

Two independent reviewers (L.L. and R.Y.Q.) screened titles and abstracts on Rayyan systematic review software.^17^ We manually excluded duplicate articles. Both reviewers subsequently completed full-text review independently and in duplicate. Discrepancies in judgment within screening stages were resolved by a third reviewer (R.Q.M.).

#### Data Extraction and Analysis

We developed a data extraction form, which was piloted on a subset of included studies. The data form extracted the following characteristics: author name, publication year, study country, study design, participant characteristics, intervention and control protocols, and outcome data. Reviewers (L.L., R.Y.Q., R.Q.M.) performed data extraction independently and in duplicate on Google Sheets (Alphabet Inc., United States). The reviewers discussed any discrepancies until a consensus was reached. We calculated descriptive statistics, including means, standard deviations, counts, proportions, and ranges using Google Sheets. Meta-analyses were not performed due to heterogeneity in outcomes and reporting of statistics.

#### Assessment of Quality

Two reviewers independently assessed the quality of included studies using the Quality Assessment Tool for Studies with Diverse Designs (QATSDD). The QATSDD is a validated 16-criterion instrument used to measure the methodological quality of studies with quantitative, qualitative, and mixed method designs. The scores for each item include: 0 (not reported), 1 (slightly reported), 2 (moderately reported), 3 (completed reported). Maximum scores are 42 for qualitative and quantitative studies, and 48 for mixed methods studies. Validity and reliability of the QATSDD have previously been established.^18^

## Results

### Search Results

**Figure 1.**
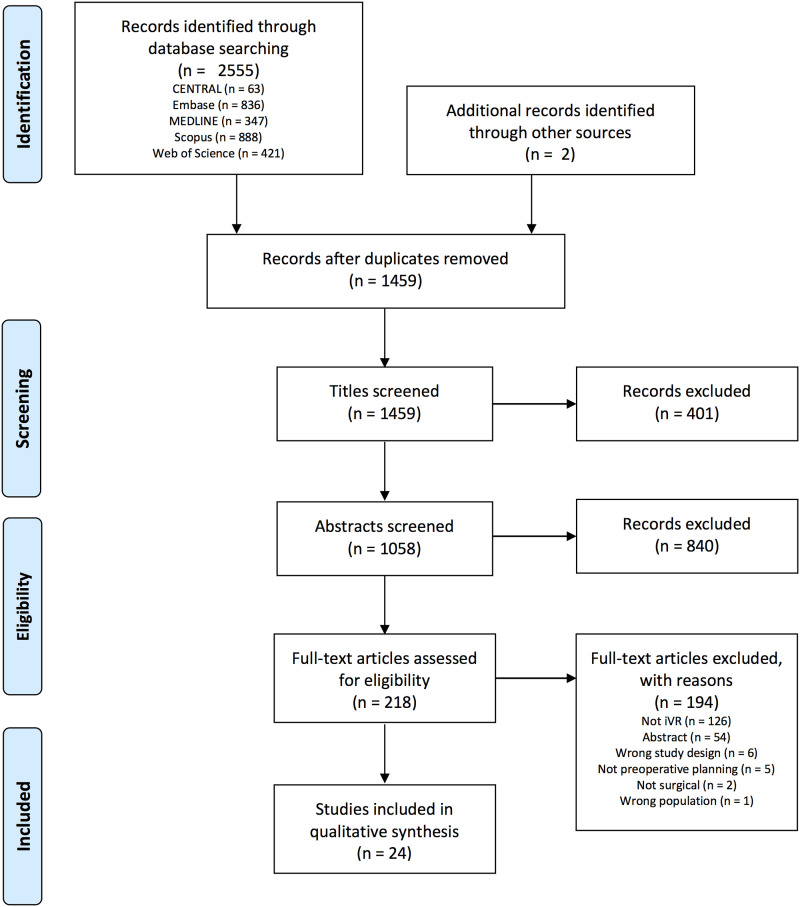
PRISMA flow diagram.

The systematic search yielded 2,555 studies in total, with two additional studies found through hand searching and review of references. After duplicate removal, 1,459 titles were screened. From review of full texts, 24 articles were included for qualitative synthesis (see Figure 1 for full PRISMA flow diagram). The κ score for interrater agreement at the title, abstract, and full-text stages were .72 (substantial agreement), .81 (almost perfect agreement), and .93 (almost perfect agreement) respectively.^19^

### Study Characteristics

Table 1 depicts the main study characteristics. Study types included randomized controlled trials (RCT; 1/24; 4%),^29^ historical control trials (3/24; 13%),^25,28,34^ prospective studies (7/24; 29%),^37,38,40–42^ combined prospective study and case report (1/24; 4%),^39^ cross-sectional studies (5/24; 21%),^23,31,33,43^ case series (3/24; 13%),^20,24,36^ and case reports (4/24; 17%).^21,22,32,35^ Within comparative studies, control interventions included CT or MRI viewed on 2D screens,^25,28,29,31,34,37–42^ 3D reconstructions of CT or MRI scans viewed on 2D screens,^27^ 3D printed models,^27^ and no preoperative planning.^43^

**Table 1.** Study Characteristics (n = 24).

Study	Country	Study Design	Procedure (discipline)	iVR Simulator (software, headsets, imaging input)	Participants (n)		QATSDD Score
					Patients	Surgeons	
Croci et al., 2020^20^	Switzerland	Case series	Spine surgery (orthopedic surgery)	SpectoVR (Diffuse Inc., Switzerland), HTC Vive, CT/MRI	8	NR	7/42 (16.7%)
Giacalone et al., 2019^21^	Belgium	Case report	Lymphovenous anastomosis (plastic surgery)	Medicalholodeck (Switzerland), HTC Vive, SPECT-CT + lymphoscintigraphy	1	NR	4/42 (9.5%)
González Romo et al., 2020^22^	Chile	Case report	ACA aneurysm clipping (neurosurgery)	Sketchfab (France), VR One Plus (Zeiss, Germany) + smartphone, CT	1	1	7/42 (16.7%)
Kenngott et al., 2021^23^	Germany	Cross-sectional	Extended right hemihepatectomy (general surgery)	IMHOTEP (Karlsruhe Institute for Technology, Germany), HTC Vive, CT	1	105 (57 medical students, 35 residents, 13 attending surgeons)	17/42 (40.5%)
Ong et al., 2018^24^	United States	Case series	Congenital heart disease repair (cardiothoracic surgery)	DICOM to Print (3D Systems, United States), HTC Vive, CT	2	4	4/42 (9.5%)
Parkhomenko et al., 2019^25^	United States	Historical control trial	Percutaneous nephrolithotomy (urology)	Bosc (Pyrus Medical, United States), Oculus Rift (Facebook Inc., United States), CT	iVR: 25 Control: 25	4	17/42 (40.5%)
Sadeghi et al., 2020^26^	Netherlands	Cross-sectional	Cardiac surgery (cardiothoracic surgery)	CardioVR (MedicalVR, Netherlands), Oculus Rift, CT	6	5	17/48 (35.4%)
Sampogna et al., 2017^27^	Italy	Prospective study	Abdominal tumour resection (general surgery/urology)	Unity 3D (Unity Technologies, United States), Oculus Rift, CT/MRI	15	20	13/42 (31.0%)
Shirk et al., 2019a^28^	United States	Historical control trial	Robotic-assisted partial nephrectomy (urology)	Unspecified smartphone app, Google Cardboard (Alphabet Inc., United States) + smartphone, CT/MRI	iVR: 30 Control: 30	3	24/42 (57.1%)
Shirk et al., 2019b^29^	United States	RCT	Robotic-assisted partial nephrectomy (urology)	Reveal (Ceevra Inc., United States), Google Cardboard + smartphone, CT/MRI	iVR: 44 Control: 48	11	31/42 (73.8%)
Sugiyama et al., 2021^30^	Japan	Prospective study	Cerebrovascular surgeries (neurosurgery)	BananaVision (Colorado State University, United States), Samsung Odyssey (Korea), CTA	18	14 (10 residents, 1 fellow, 3 experts)	16/48 (33.3%)
Timonen et al., 2020^31^	Finland	Cross-sectional (cadaveric)	Temporal bone surgery (otolaryngology)	SurgeryVision (Adesante, Finland), HTC Vive Pro, CT	5	5	24/48 (50.0%)
Ujiie et al., 2021^32^	Japan	Case report	VATS sublobar resection (cardiothoracic surgery)	BananaVision, Samsung Odyssey, CT	1	NR	7/42 (16.7%)
Vertemati et al., 2019^33^	Italy	Cross- sectional	Laparoscopic partial nephrectomy/nonspecific (general surgery)	Unity 3D, Samsung Gear (Korea) + smartphone, CT/MRI	1	22 (15 PGY2-3 residents, 7 attending surgeons)	16/42 (38.1%)
Xie et al., 2021^34^	United States	Historical control trial	Laparoscopic donor nephrectomy (urology)	Bosc (Pyrus Medical, United States), Oculus Rift, CT	iVR: 20 Control: 45	2	18/42 (42.9%)
Yamada et al., 2019^35^	Japan	Case report	Partial nephrectomy (urology)	Holoeyes (Japan), Mirage Solo (Lenovo Group Limited, Hong Kong), CT	1	1	2/42 (4.8%)
Yan et al., 2020^36^	United States	Case series	Pediatrics MCA aneurysm procedures (neurosurgery)	SuRgical Planner (Surgical Theater, United States), Oculus Rift, CT	5	NR	8/42 (19.0%)
Zawy Alsofy et al., 2021a^37^	Germany	Prospective study	MIS vs Open Dorsal spinal fusion (neurosurgery)	3D Slicer (SurgicalPlanning Laboratory, United States), HTC Vive (HTC Corporation, Taiwan), CT/MRI	12	12	15/42 (35.7%)
Zawy Alsofy et al., 2021b^38^	Germany	Prospective study	Meningioma resection (neurosurgery)	3D Slicer, HTC Vive, CT-CTA/MRI	30	10	18/42 (42.9%)
Zawy Alsofy et al., 2021c^39^	Germany	Prospective study + case report	Microvascular decompression of trigeminal neuralgia (neurosurgery)	3D Slicer, HTC Vive, MRI	24	10	19/48 (39.6%)
Zawy Alsofy et al., 2020a^40^	Germany	Prospective study	Deep infratentorial tumour resection (neurosurgery)	3D Slicer, HTC Vive, MRI	14	9	13/42 (31.0%)
Zawy Alsofy et al., 2020b^41^	Germany	Prospective study	ACA aneurysm clipping (neurosurgery)	3D Slicer, HTC Vive, CTA	26	10	18/42 (42.9%)
Zawy Alsofy et al., 2019^42^	Germany	Prospective Study	Cervical spine decompression (neurosurgery)	3D Slicer, HTC Vive, CT	10	12	15/42 (35.7%)
Zhou et al., 2019^43^	China	Cross-sectional (cadaveric)	Percutaneous endoscopic discectomy (orthopedic surgery)	Software and headset unspecified, CT	12	4	11/42 (26.2%)

Abbreviations: CT = computed tomography; CTA = computed tomography angiogram; MRI = magnetic resonance imaging; PGY = post-graduate year; QATSDD = quality assessment tool for studies with diverse designs; RCT = randomized controlled trial; SPECT = single-photon emission computerized tomography.

The majority of studies were conducted in the discipline of neurosurgery (10/24; 42%),^22,30,36–42^ with the remaining studies conducted in urology (5/24; 21%),^25,28,29,34,35^ cardiothoracic surgery (3/24; 13%),^24,26,32^ general surgery (2/24; 8%),^23,33^ orthopedic surgery (2/24; 8%),^20,43^ plastic surgery (1/24; 4%),^21^ and combined general surgery and urology (1/24; 4%).^27^ In total, 264 medical personnel were included across all studies, with the exception of three studies,^20,21,36^ which did not report the number of surgeons who participated. Of these, 146/264 (55%) were attending surgeons, 1/264 (<1%) was a fellow, 60/264 (23%) were residents, and 57/264 (22%) were medical students. There were a total of 460 patients whose data were rendered for preoperative planning, including 17 cadaveric specimens.

### Methodological Quality

The mean ± standard deviation (SD) QATSDD score of included studies was 32.9 ± 16.1%. Table 2 breaks down mean QATSDD scores by domain. The highest-scoring domain was “statement of aims/objectives in main body of report” (2.3 ± .9 out of 3), and the lowest-scoring domains were “evidence of user involvement in design” (.1 ± .4 out of 3) for all study designs, and “assessment of reliability of analytical process” (.0 ± .0 out of 3) for qualitative studies.

**Table 2.** QATSDD Scores.

Domain	Mean Score (0-3)	Standard Deviation
All Studies (n = 24)		
Explicit theoretical framework	1.6	0.7
Statement of aims/objectives in main body of report	2.3	0.9
Clear description of research setting	1.4	0.8
Evidence of sample size considered in terms of analysis	.25	0.8
Representative sample of target group of a reasonable size	1.0	0.8
Description of procedure for data collection	1.2	0.9
Rationale for choice of data collection tool(s)	0.2	0.6
Detailed recruitment data	.75	0.5
Fit between research question and method of analysis	1.8	1.3
Good justification for analytical method selected	0.2	0.7
Evidence of user involvement in design	0.1	0.4
Strengths and limitations critically discussed	1.2	0.8
Quantitative and Mixed Methods Studies (n = 16)		
Statistical assessment of reliability and validity of measurement tool(s)	.75	1.1
Fit between stated research questions and method of data collection	1.8	0.5
Qualitative and Mixed Methods Studies (n = 12)		
Fit between stated research question and format and content of data collection tool	0.9	0.5
Assessment of reliability of analytical process	0.0	0.0
Total Score (%, mean±SD): 32.9±16.1		

Abbreviations: SD = standard deviation; QATSDD = quality assessment tool for studies with diverse designs.

### Technology Use

The included studies used a wide array of software, hardware, and imaging input options (Table 1). Most studies used standalone head-mounted devices, including HTC Vive (HTC Corporation, Taiwan; 11/24),^20,21,23,24,31,37–42^ Oculus Rift (Facebook Inc., United States; 5/24),^25–27,34,36^ Samsung Odyssey (Korea; 2/24),^30,32^ and Mirage Solo (Lenovo Group Limited, Hong Kong; 1/24).^35^ Headsets using smartphone inputs were also used, including Google Cardboard (Alphabet Inc., United States; 2/24),^28,29^ VR One Plus (Zeiss, Germany; 1/24),^22^ and Samsung Gear (Korea; 1/24).^33^ The hardware used in one study was not specified.^43^ CT or MRI scans served as the input image source for all studies, although one study additionally incorporated lymphoscintigraphy.^21^

To visualize patient-specific cross-sectional imaging in iVR, authors most commonly loaded CT or MRI imaging files in the common Digital Imaging and Communications in Medicine (DICOM) format into a commercially-available segmentation software. Segmentation manually or automatically separated adjacent anatomical structures into discrete objects (e.g. separate renal tumour from renal parenchyma). Depending on the software, the post-segmentation file then underwent additional processing or was directly loaded onto iVR visualization software. Software functions included interaction (e.g. moving, cutting, erasing) with models through handheld controllers, increasing the transparency of certain structures to view underlying anatomy, and multiuser modalities.

### Patient-specific Outcomes

Patient-related outcome measures analyzed included operative time, blood loss, fluoroscopy time, complications, and length of stay. Only studies reporting quantitative results are discussed. Table 3 summarizes key methods and findings.

**Table 3.** Patient-Related Outcomes: Summary of Results Following Preoperative Planning With iVR, Arranged by Study Design.

Study	Study Design	Description of Outcome Measurement	Comparator	Favours iVR or control
Operative Time (Quantitative; n = 4)				
Shirk et al., 2019b^29^	RCT	Review of intraoperative records	CT or MRI alone	Equivalent
Parkhomenko et al., 2019^25^	Historical control trial	Review of intraoperative records	CT alone	Equivalent
Shirk et al., 2019a^28^	Historical control trial	Review of intraoperative records	CT or MRI alone	Equivalent
Xie et al., 2021^34^	Historical control trial	Review of intraoperative records	CT alone	iVR
Blood Loss (Quantitative; n = 4)				
Shirk et al., 2019b^29^	RCT	Review of intraoperative records	CT or MRI alone	Equivalent
Parkhomenko et al., 2019^25^	Historical control trial	Review of intraoperative records	CT alone	iVR
Shirk et al., 2019a^28^	Historical control trial	Review of intraoperative records	CT or MRI alone	Equivalent
Xie et al., 2021^34^	Historical control trial	Review of intraoperative records	CT alone	Equivalent
Fluoroscopy Time (Quantitative; n = 2)				
Parkhomenko et al., 2019^25^	Historical control trial	Review of intraoperative records	CT alone	iVR
Zhou et al. 2019^43^	Cross-sectional (cadaveric)	Records from simulated cadaveric operation	No preoperative planning	iVR
Complications (Quantitative; n = 4)				
Parkhomenko et al., 2019^25^	Historical control trial	Review of intraoperative records	CT alone	Equivalent
Shirk et al., 2019a^28^	Historical control trial	Review of intraoperative records	CT or MRI alone	Equivalent
Xie et al., 2021^34^	Historical control trial	Review of intra- and postoperative records	CT alone	iVR
Sugiyama et al., 2021^30^	Prospective study	Review of postoperative records	None	ND
Length of Stay (Quantitative; n = 3)				
Shirk et al., 2019b^29^	RCT	Review of intraoperative records	CT or MRI alone	Equivalent
Shirk et al., 2019a^28^	Historical control trial	Review of intraoperative records	CT or MRI alone	Equivalent
Xie et al., 2021^34^	Historical control trial	Review of intraoperative records	CT alone	Equivalent

Abbreviations: CT = computerized tomography; iVR = immersive virtual reality; ND = not determinable (cannot determine superiority of one intervention) MRI = magnetic resonance imaging; RCT = randomized controlled trial.

#### Operative Time

Operative time was reported in four controlled trials, with findings in favour of iVR in one historical control trial,^34^ and equivalent results in the three remaining studies.^25,28,29^ In the study demonstrating significant differences between groups, patients were prospectively recruited to receive iVR preoperative planning for laparoscopic donor nephrectomy, and compared with retrospectively-matched controls whose operations were planned with 2D CT imaging only. The median (interquartile range; IQR) operative time was 191 (58) minutes in the iVR group, and 241 (113) minutes in the control group (*P* < .001).^34^ Patients underwent robotic-assisted partial nephrectomies in two other studies. One found the operative times to be 172.6 ± 48.5 minutes for the iVR group and 173.3 ± 49.6 minutes for the conventional CT or MRI group respectively (P = .70).^29^ The second study was a historical control trial that measured mean operative times of 168 minutes in the iVR group and 188 minutes in the control group (*P* = .12). After results were back-transformed from linear regression controlling for nephrotomy score, surgeon, and resident involvement, mean operative times were 141 in the iVR group and 201 in the control group (*P* < .0001).^28^ The last historical control trial found that the median operative time was 155 minutes in the iVR group and 180 minutes in the control group (*P* = .19).^25^

#### Blood Loss

Intraoperative blood loss was reported in four controlled trials, with positive findings in favour of iVR in one historical trial,^25^ and equivalent results in the three remaining studies.^28,29,34^ The positive historical control trial found that median blood loss during percutaneous nephrolithotomy was 50 mL for iVR planning patients, and 100 mL for conventional preoperative planning patients (*P* < .01).^25^ In another RCT, the mean estimated blood loss was 124.5 ± 90.5 mL in the iVR group and 145.7 ± 140.4 mL in the control group (*P* = .71) for robotic partial nephrectomy. In a historical control trial for the same procedure, the iVR group sustained a mean estimated blood loss of 135 mL and the control group sustained an estimated blood loss of 150 mL (*P* = .67). In this study, blood loss was significantly different after results were back transformed from a linear regression controlling for nephrotomy score and surgeon with mean blood losses of 133 mL vs 259 mL in the iVR vs control groups (*P* = .023).^28^ The last historical control trial in patients undergoing laparoscopic donor nephrectomy found no significant differences between iVR and control groups with median (IQR) blood losses of 30 (30) mL and 50 (39) mL respectively (*P* = .40).^34^

#### Fluoroscopy Time

Both studies that measured fluoroscopy time recorded positive results in favour of iVR.^25,43^ In a historical control trial with patients undergoing percutaneous nephrolithotomy, the mean (IQR) fluoroscopy time was lower in the iVR group (180 [122] seconds) vs control group (226 [296] seconds; *P* < .01).^25^ Surgeons performed transforaminal percutaneous endoscopic discectomies in cadavers in a cross-sectional study. Surgeons first performed the procedure with no preoperative planning nor intraoperative guidance on the left side, then performed the same procedure with iVR preoperative planning and isocentric navigation on the right side. Fluoroscopy times were significantly lower at all spinal levels reported (L3-L4, L4-L5, and L5-S1) when iVR plus navigation were used. For instance, the mean fluoroscopy time at the L3-L4 level was 14.64 ± 1.60 seconds and 17.21 ± 2.91 seconds (*P* = .025) in the iVR and control groups respectively.^43^

#### Complications

One study found a significantly lower post-laparoscopic donor nephrectomy 30-day complication rate (Clavien-Dindo grade I and above) in the iVR group (2/20; 10%) compared to control (10/45; 22%; *P* < .001).^34^ Two historical control trials found no significant differences between iVR and control groups. The first reported an intraoperative complication rate of 4% for both groups undergoing percutaneous nephrolithotomy,^25^ and the second reported no intraoperative complications in the iVR group and two (7%) complications in the control group for robot-assisted partial nephrectomies (*P* = .49).^28^ Patients undergoing cerebrovascular surgery with iVR preoperative planning in one prospective study achieved a 94.4% favourable outcome rate three to four months after surgery. However, there was no comparator group, so results cannot be attributed to preoperative planning.^30^

#### Length of Stay

Three studies demonstrated no significant effect of iVR on post-operative length of stay.^28,29,34^ Of patients who underwent robotic-assisted partial nephrectomy, 53% of those whose procedures were planned with iVR and 73% of patients who underwent standard planning remained hospitalized for greater than two days (*P* = .11).^28^ A second study on the same procedure reported that 9% of patients in the iVR group and 15% of controls remained in-hospital for greater than two days (*P* = .42).^29^ The median length of stay in a final study on laparoscopic donor nephrectomy patients was two days in both groups.^34^

### Surgeon-specific Outcomes

Surgeon-specific outcome measures included impact of iVR on surgical strategy, visualization of anatomy, validity and reliability, impact on surgeon confidence, and feasibility. Table 4 summarizes key methods and findings.

**Table 4.** Surgeon-Related Outcomes: Summary of Results Following Preoperative Planning With iVR, Arranged by Study Design.

Study	Study Design	Description of Outcome Measurement	Comparator	Findings favour iVR or control
Impact on Surgical Strategy (n = 12)				
Parkhomenko et al., 2019^25^	Historical control trial	Participants asked postoperatively if iVR altered the operative approach	CT alone	Equivalent
Xie et al., 2021^34^	Historical control trial	Participants asked pre-operatively to rate agreement with statement that iVR altered surgical plan	CT alone	iVR
Sampogna et al., 2017^27^	Prospective study	Participants rated whether interventions changed surgical strategy compared to 2D imaging	3D printing, 3D reconstructions of CT or MRI displayed on 2D screens	Equivalent
Sugiyama et al., 2021^30^	Prospective study	Participants asked preoperatively to rate if iVR impacted major and minor surgical decisions	None	iVR
Zawy Alsofy et al., 2021a^37^	Prospective study	Participants asked to choose surgical approach after viewing 2D imaging, then re-surveyed after viewing iVR	CT and MRI alone	iVR
Zawy Alsofy et al., 2021b^38^	Prospective study	Participants asked to choose surgical approach after viewing 2D imaging, then re-surveyed after viewing iVR	CT or MRI alone	iVR
Zawy Alsofy et al., 2021c^39^	Prospective study + case report	Participants asked to choose surgical approach after viewing 2D imaging, then re-surveyed after viewing iVR	MRI alone	iVR
Zawy Alsofy et al., 2020a^40^	Prospective study	Participants asked to choose surgical approach after viewing 2D imaging, then re-surveyed after viewing iVR	MRI alone	iVR
Zawy Alsofy et al., 2020b^41^	Prospective study	Participants asked to choose surgical approach after viewing 2D imaging, then re-surveyed after viewing iVR	CTA alone	iVR
Zawy Alsofy et al., 2019^42^	Prospective Study	Participants asked to choose surgical approach after viewing 2D imaging, then re-surveyed after viewing iVR	CT alone	iVR
Sadeghi et al., 2020^26^	Cross-sectional	Qualitative description of experiences	None	iVR
Yan et al., 2020^36^	Case series	Qualitative description of experiences	None	ND
Visualization of Anatomy (n = 12)				
Parkhomenko et al., 2019^25^	Historical control trial	Participants surveyed pre- and postoperatively to self-rate understanding of patient and pathology anatomy	CT alone	iVR
Xie et al., 2021^34^	Historical control trial	Participants surveyed pre- and postoperatively to compare anatomy understanding with iVR vs CT	CT alone	iVR
Sugiyama et al., 2021^30^	Prospective study	Participants surveyed preoperatively to determine if iVR increased understanding of patient anatomy, and their illustrations of patient anatomy were compared with actual surgical videos	None	iVR
Zawy Alsofy et al., 2021b^38^	Prospective study	Participants asked to rate sufficiency of anatomic structure detection after viewing 2D imaging, then resurveyed after viewing iVR	CT or MRI alone	iVR
Zawy Alsofy et al., 2021c^39^	Prospective study + case report	Participants asked to identify pathology after viewing 2D imaging, then resurveyed after viewing iVR	MRI alone	iVR
Zawy Alsofy et al., 2020b^41^	Prospective study	Participants asked to rate sufficiency of anatomic structure detection after viewing 2D imaging, then resurveyed after viewing iVR	CTA alone	iVR
Sampogna et al., 2017^27^	Prospective study	Participants were surveyed to compare comprehension of anatomy compared to 2D imaging	3D printing, 3D reconstructions of CT or MRI displayed on 2D screens	Equivalent
Sadeghi et al., 2020^26^	Cross-sectional	Participants surveyed postoperatively to see if iVR allowed for more accurate anatomy review compared to conventional CT, and qualitative description of experiences	None	iVR
Timonen et al., 2020^31^	Cross-sectional (cadaveric)	Participants rated anatomic visualization and understanding using a survey	CT alone	iVR
Croci et al., 2020^20^	Case series	Qualitative description of experiences	None	ND
Ong et al., 2018^24^	Case series	Qualitative description of experiences	None	ND
Yan et al., 2020^36^	Case series	Qualitative description of experiences	None	ND
Validity and Reliability (n = 2)				
Timonen et al., 2020^31^	Cross-sectional (cadaveric)	Participants asked to subjectively rate face validity and content validity. Reliability and criterion validity were established by comparing virtual with physical cadaveric measurements	CT alone	iVR
Sadeghi et al., 2020^26^	Cross-sectional	Criterion validity was established by comparing virtual with intraoperative measurements	None	ND
Impact on Surgeon Confidence (n = 3)				
Parkhomenko et al., 2019^25^	Historical control trial	Participants asked preoperatively if iVR improved understanding and confidence for surgery	CT alone	iVR
Xie et al., 2021^34^	Historical control trial	Participants asked preoperatively to rate confidence in understanding patient anatomy	CT alone	iVR
Yan et al., 2020^36^	Case series	Qualitative description of experiences	None	ND
Feasibility (Quantitative; n = 4)				
Kenngott et al., 2021^23^	Cross-sectional	Participants asked to rate iVR’s potential for clinical use, and to predict number of years until daily clinical use	None	Equivalent
Sadeghi et al., 2020^26^	Cross-sectional	Participants asked postoperatively to rate agreement with statements on future use of VR	None	iVR
Timonen et al., 2020^31^	Cross-sectional (cadaveric)	Participants asked to rate feasibility of inclusion into clinical surgical planning	CT alone	Equivalent
Vertemati et al., 2019^33^	Cross-sectional	Participants asked to rate iVR ease of use (feasibility)	None	iVR

Abbreviations: CT = computerized tomography; iVR = immersive virtual reality; ND = not determinable (cannot determine superiority of one intervention) MRI = magnetic resonance imaging; RCT = randomized controlled trial; VR = virtual reality.

#### Impact on Surgical Strategy

The impact of preoperative iVR use on surgeons’ surgical plans was assessed in 12 studies. Of these, nine demonstrated results favouring iVR,^26,30,34,37–42^ two reported equivalent outcomes,^25,27^ and one used a case series design which did not allow for determination of superiority.^36^ The majority (6/9; 67%) of the nine positive studies were completed by one group who surveyed neurosurgeons on operative approaches after viewing retrospective 2D CT or MRI scans, then resurveyed the same surgeons three to four weeks later after viewing the corresponding iVR models.^37–42^ All studies showed significant changes in preferred surgeon operative approaches. Surgeons in two studies viewed conventional cross-sectional imaging, then corresponding iVR models. In one study, surgeons strongly agreed (median [IQR] = 5 [2-5] out of 5) that “evaluation of the [iVR] model altered [their] preoperative surgical plan.”^34^ In another study, 61.1% and 27.8% of participants rated iVR as effective to enhance decision-making for minor and major surgical techniques respectively, with more favourable ratings among trainees compared to experts (P < .05).^30^ The last positive study used a qualitative approach, identifying the impact of iVR on surgical strategy in six cardiac surgery procedures.^26^ In one study with equivalent results, 40% of surgeons who viewed conventional CT scans and iVR models agreed that iVR altered the surgical approach.^25^ Surgeons in the second study compared iVR, 3D printed models, and 3D CT or MRI reconstructions viewed on flat screens with conventional CT or MRI. Mean agreement that iVR, 3D printed models, and 3D reconstructions changed surgical strategy compared to 2D imaging was 3.7, 3.9, and 4.1 respectively (out of 5, with 5 indicating greatest agreement).^27^

#### Visualization of Anatomy

Eight of 12 studies reported improved anatomy visualization with iVR use.^25,26,30,31,34,38,39,41^ Table 4 describes the methodology used in each study, with most involving subjective ratings. Two studies objectively verified anatomy understanding. Surgeons could not identify the affected side in patients with trigeminal neuralgia 7% of the time using conventional MRI, but only 2% of the time with iVR, with *P* = .005 for pathology localization overall.^39^ When asked to illustrate schematics of patients’ cerebral aneurysms, accuracy scores improved significantly (*P* < .05) after viewing iVR models compared to surgical video. When surveyed, surgeons in this study agreed that iVR was effective for increasing understanding of patient-specific anatomy in 83.3% of cases, with trainees more likely to “strongly agree” than experts (*P* < .01).^30^ In a study with equivalent results, surgeons rated their agreement that iVR, 3D printing, and 3D reconstructions of CT or MRI allowed for better visualization of anatomic relationships as compared to traditional 2D imaging as 4.3, 4.4, and 3.7 out of 5 respectively.^27^ The remaining three studies were case series which qualitatively described how iVR could improve anatomy detection.^20,24,36^

#### Validity and Reliability

Two studies validated patient-specific iVR models.^26,31^ Attendings in one study were asked to rate iVR models and conventional 2D imaging on various domains of face and content validity using a 5-point Likert scale. Mean scores were significantly higher for iVR compared to 2D imaging in both face (3.78 ± .83 vs 3.20 ± .99, *P* = .002) and content validity (4.33 ± .62 vs 3.23 ± .62, *P* < .001). Moreover, criterion validity was established by comparing distance measurements using imaging vs real cadavers. Immersive VR measurements deviated an average of .815 ± .665 mm from real measurements, while 2D imaging measurements deviated 1.753 ± 3.563 mm (*P* = .065). The intraclass correlation coefficient for interrater reliability of iVR measurements was >.95.^31^ Criterion validity was weakly established in a case series when the left atrial appendage of a patient was measured to be 28 mm using iVR, and 30 mm intraoperatively.^26^

#### Impact on Surgeon Confidence

Two historical control trials surveyed surgeons on confidence after iVR use, both finding iVR improved confidence and understanding compared to conventional CT scans alone.^25,34^ Qualitatively, iVR improved preoperative confidence in one case series.^36^

#### Feasibility

Four cross-sectional studies asked users to rate the feasibility of iVR incorporation into regular preoperative practice.^23,26,31,33^ Results were mixed: two studies demonstrated good feasibility,^26,33^ and two studies were equivalent.^23,31^ Notably, one study found that feasibility was rated lower with advancing stages of surgical training. When asked to rate potential for clinical use, positive responses (indicated by mean scores ≥4/5 on a Likert scale) were achieved in 87.7% of medical students, 64.7% of residents, and 69.2% of attendings (*P* = .035 between training levels). Upon further breakdown, medical students, residents, and staff gave positive responses 84.2%, 85.7%, and 76.9% of the time when asked about potential for medical student training and 85.7%, 76.5%, and 69.2% when asked about potential for resident training, respectively. Predicted time until implementation for daily use was lowest in residents at 4.26 years, and greatest in staff at 4.28 years.^23^ In a different study, residents rated feasibility lower than experts on a 5-point scale (3.93 ± 1.163/5 and 4.71 ± .756 respectively). Utility for teaching medical students was rated more highly than utility for teaching residents by both residents and experts.^33^

### Resource Use

#### Cost

Some studies reported costs and resources required to produce iVR models. Hardware expenses were estimated at 1586 USD,^34^ 1700 USD,^25^ and 4000 to 6000 EUR (approximately 4800 to 7100 USD).^26^ Two studies used Google Cardboard, a 15 USD low-tech headset that pairs with smartphones.^28,29^ Other resource considerations include the necessary expertise to perform image segmentation or to operate iVR software.^23,26^ One center did not incur additional personnel expenses to run iVR software,^25^ whereas another center implemented a dedicated 3D-surgery team consisting of medical and technical staff.^26^

#### Time

The duration of iVR model production was also reported. Mean production times were reported to be less than two minutes,^30^ 9 ± 4 minutes,^38^ 15 minutes,^26^ and one to two hours.^21,25,27,33,34^ Often, production time decreased with greater attempts.^25,27,34,38,39^ For instance, task duration decreased from six to ten hours to one to two hours after creation of the first three to five models.^34^ The reasonableness of time to obtain iVR, 3D-printed, and 3D-rendered models was rated 4.0/5, 3.8/5, and 4.2/5 respectively.^27^

## Discussion

Immersive VR is a novel medium that allows surgeons to manipulate realistic patient-specific 3D models for surgical planning. Short-term patient-specific outcomes such as operative time, blood loss, complications, and length of stay do not differ significantly between patients whose procedures were planned with iVR vs those in whom only conventional CT or MRI planning were used. Fluoroscopy time, reported in two studies, may be decreased with preoperative use of iVR.^25,43^ Only six studies (27.2%) representing 287 patients reported on objective clinical outcomes, while the majority of the included studies investigated surgeon-specific outcomes. Although there is a lack of evidence supporting iVR’s impact on objective clinical outcome measures, its utility is rated favourably among surgeons. Of the surgeon-specific outcomes, iVR positively impacted surgical strategy, visualization of anatomy, and surgeon confidence. Over time, these outcomes can improve patient selection, contribute to developments in surgical technique, and promote collaboration among colleagues.^44^ Validity, reliability, and feasibility of preoperative iVR were also assessed, with limited but favourable evidence. The majority of surgeon-specific outcomes were assessed using ad hoc surveys or unstructured qualitative feedback. Due to the heterogeneity in outcome measurement and reporting, cross-study comparisons and statistical pooling or analysis could not be completed.

Neurosurgery and urology were highly represented in included studies, reflecting the ability of CT and MRI to capture relevant anatomy. Immersive VR has been used to replicate a variety of different tissue types, ranging from soft tissue structures (e.g., heart, pancreas, liver, brain, vessels, kidneys) to hard tissue structures (e.g., skull, spine). However, iVR visualizations are dependent on the quality of the input data.^25,27,34,37,38^ In a study by Zawy Alsofy and colleagues, the anatomy of small cerebral branches and perforators were missing in the iVR model.^41^ Moreover, soft tissue layers such as skin and muscle have not yet been reproduced.^22^ Certain abdominal organs (e.g., small and large intestines) are difficult to image in detail as they can show variable voxel intensities and shapes due to their solid, liquid and air components, and thus also require expensive manual segmentation.^33^

Similar to costs reported in the literature, the costs for iVR hardware included in this study ranged from 15 USD to 7100 USD.^12,45^ This is comparable to the costs of 3D printing, where printers can cost anywhere between 1000 to 2200 USD and each 3D printed model ranges from two to 330 USD.^7^ In the studies reviewed, iVR model production time ranged from less than two minutes to two hours. This starkly contrasts the time required to produce 3D printed models, which can range from five to 72 hours.^3,7^ An obstacle for the widespread implementation of iVR in preoperative planning lies in expertise and personnel requirements to produce 3D segmentations of CT and MRI images.^22,23,26^ However, as iVR and imaging technologies evolve, it is possible that the realism of iVR models will increase, while costs, production times and personnel requirements will be reduced.

### Future Directions

Immersive VR is a nascent technology that presents as an exciting supplement to preoperative planning without exhausting financial or time resources. However, its full capabilities have yet to be defined. A number of iVR simulators included in this study (e.g., Bananavision,^30,32^ Medicalholodeck Cloud^21^) support multi-user modes, which can facilitate team-based preoperative planning as well as surgical training.^21,30,32,46^ Unfortunately, none of the included studies investigated this function.

Immersive VR has previously been shown to be an effective surgical training simulator.^12^ Here, preoperative iVR was generally rated to be more favourable for trainees than attendings. Effectiveness for understanding patient-specific anatomy,^30,33^ impact on decision-making,^30^ and utility for teaching^33^ were greater for more junior surgeons. This is consistent with previous demonstrating that 3D reconstructions improve resident understanding of patient anatomy compared to 2D cross-sectional views, resulting in more accurate surgical plans.^4^ The cognitive load required to interpret cross-sectional imaging is known to be remarkably high, especially for novices, which can distract from surgical planning and learning.^5^ In the future, patient-specific preoperative planning and surgical training functions of iVR can be merged so that procedures can be rehearsed in a higher fidelity environment with larger theoretical advantages on patient outcomes. Future work should also further clarify how outcomes differ by surgeon experience to understand the role of preoperative iVR in training and surgical planning in novices.

With the majority of included studies being small scale observational studies, there is a lack of high quality studies that assess the use of iVR for preoperative planning within larger patient and surgeon populations over an extended follow-up period. This may help to elucidate the effects of improved surgeon preparedness and confidence on the longer-term patient specific outcomes. Moreover, there is substantial heterogeneity in the outcomes and outcome measures used to assess the effectiveness of the technology as an intervention. There is a need for the use of standardized outcomes and validated instruments within high quality randomized trials to investigate the efficacy iVR on preoperative planning before implementation into practice. There is also value in further exploring facilitators and barriers to implementation using robust qualitative methodology.

### Limitations

This review has several limitations. Our initial search was restricted to English-language articles, limiting the scope of included articles. Furthermore, a meta-analysis could not be completed due to heterogeneity within outcome measures. The quality of included studies limited the strength of conclusions. Although considered the lowest level of evidence, case studies and case series were included to ensure all iVR applications in preoperative planning were considered.^47^ Methodological improvements should be made in future work. Within qualitative studies, validated methodology for data collection and analysis should be utilized. Comparative studies should aim to randomize group allocation and blind participants and assessors where possible. In addition, the crossover effect should be minimized, as multiple included studies instructed surgeons to use conventional cross-sectional imaging first, followed by iVR. Within all studies, participant recruitment should be transparently reported, and previously validated tools should be used to measure outcomes.

## Conclusion

Immersive VR is a budding technology that can improve preoperative planning in the digital era. Although preoperative planning with iVR was shown to minimally impact short-term patient-specific outcomes (e.g., operative time, blood loss, complications, and length of stay) with a potential decrease in fluoroscopy time compared to conventional imaging, it can alter surgical technique, improve anatomy visualization, and is rated favourably by surgeons. The current body of evidence is restricted by low quality and heterogeneous studies, limiting the conclusions that can be drawn. As such, further high quality studies must be conducted to fully elucidate the long-term global effect of iVR for preoperative planning on patient- and surgeon-specific outcomes. The greatest potential for preoperative planning with iVR may lie in its integration with surgical training functions.

## Supplemental Material

Supplemental Material - Immersive Virtual Reality for Patient-specific Preoperative Planning: A Systematic Review Click here for additional data file. Supplemental Material for Immersive Virtual Reality for Patient-specific Preoperative Planning: A Systematic Review by Lucy Lan, Randi Q. Mao, Reva Y. Qiu, and Darren de Sa in Surgical Innovation.
