# Supplementary material for: Immersive Virtual Reality for Patient-Specific Preoperative Planning: A Systematic Review
Source: Surg Innov. 2022 Nov 30;30(1):109–22. doi: 10.1177/15533506221143235 (PMC9925905; doi:10.1177/15533506221143235)
Supplement: Supplemental Material - Immersive Virtual Reality for Patient-specific Preoperative Planning: A Systematic Review [file sj-pdf-1-sri-10.1177_15533506221143235.pdf]

Table S.1: Search Strategy

Database	Results	Keywords and Filters
MEDLINE	347	(virtual reality.mp. OR exp virtual reality/ OR VR.mp. OR iVR.mp. OR head-mounted.mp. OR head mounted.mp. OR face-mounted.mp. OR face mounted.mp.) AND (surgical procedures, operative.mp. OR exp surgical procedures, operative/ OR surg*.mp.) AND (preop*.mp. OR pre-op*.mp. OR pre op*.mp. OR pre-surgical.mp. OR pre-surgery.mp. OR presurgical.mp. OR presurgery.mp. OR pre surg*.mp. OR patient-specific.mp. OR patient specific.mp.) AND (plan*.mp. OR train*.mp. OR practi*.mp. OR warm-up*.mp. OR warm up*.mp.)<br>Filters applied: english language, yr="2000-Current," NOT (animal/ NOT (human/ AND animal/)), remove duplicates
EMBASE	836	(virtual reality.mp. OR exp virtual reality/ OR VR.mp. OR iVR.mp. OR head-mounted.mp. OR head mounted.mp. OR face-mounted.mp. OR face mounted.mp.) AND (surgical procedures, operative.mp. OR exp surgery/ OR surg*.mp.) AND (preop*.mp. OR pre-op*.mp. OR pre op*.mp. OR pre-surgical.mp. OR pre-surgery.mp. OR presurgical.mp. OR presurgery.mp. OR pre surg*.mp. OR patient-specific.mp. OR patient specific.mp.) AND (plan*.mp. OR train*.mp. OR practi*.mp. OR warm-up*.mp. OR warm up*.mp.)<br>Filters applied: english language, yr="2000-Current," NOT (animal/ NOT (human/ AND animal/)), remove duplicates
CENTRAL	63	([mh"virtual reality"] OR (virtual reality):ti,ab,kw OR (VR):ti,ab,kw OR (iVR):ti,ab,kw OR (head-mounted):ti,ab,kw OR (head mounted):ti,ab,kw OR (face-mounted):ti,ab,kw OR (face mounted):ti,ab,kw) AND ([mh"surgical procedures, operative"] OR (surgical procedures, operative):ti,ab,kw OR (surg*):ti,ab,kw) AND ((preop*):ti,ab,kw OR (pre-op*):ti,ab,kw OR ("pre op*"):ti,ab,kw OR (pre-surgical):ti,ab,kw OR (pre-surgery):ti,ab,kw OR (presurgical):ti,ab,kw OR (presurgery):ti,ab,kw OR ("pre surg*"):ti,ab,kw OR (patient-specific):ti,ab,kw OR ("patient specific*"):ti,ab,kw) AND ((plan*):ti,ab,kw OR (train*):ti,ab,kw OR (practic*):ti,ab,kw OR (warm-up*):ti,ab,kw OR ("warm up*"):ti,ab,kw)<br>Filters applied: publication year from 2000 to 2020, in Trials (word variations have been searched)
Web of Science	421	((TS=("virtual reality" OR VR OR iVR OR head-mounted OR "head mounted" OR face-mounted OR "face mounted") and TS=(surgical procedures, operative OR surg*) AND TS=(preop* OR pre-op* OR "pre op*" OR pre-surgical OR pre-surgery OR presurgical OR presurgery OR "pre surg" OR patient-specific OR "patient specific") AND TS=(plan* OR train* OR practi* OR warm-up* OR "warm up*"))<br>Filters applied: english language, timespan=2000-2020
Scopus	888	TITLE-ABS-KEY ("virtual reality" OR VR OR iVR OR head-mounted OR "head mounted" OR face-mounted OR "face mounted") AND TITLE-ABS-KEY ("surgical procedures,operative" OR surg*) AND TITLE-ABS-KEY (preop* OR pre-op* OR "pre op" OR pre-surgical OR pre-surgery OR

presurgical OR presurgery OR “pre surg*” OR patient-specific OR “patient specific”) AND TITLE-ABS-KEY (plan* OR train* OR practi* OR warm-up* OR “warm up*”)
Filters applied: english language, pubyear > 1999
